# The epidemiology of Moebius syndrome in Italy

**DOI:** 10.1186/s13023-021-01808-2

**Published:** 2021-04-07

**Authors:** Arturo Carta, Stefania Favilla, Giacomo Calzetti, Maria Cristina Casalini, Pier Francesco Ferrari, Bernardo Bianchi, Maria Beatrice Simonelli, Roberta Farci, Stefano Gandolfi, Paolo Mora

**Affiliations:** 1 Ophthalmology Unit, Department of Medicine and Surgery, University of Parma, Via Gramsci 14, 43125 Parma, Italy; 2 Independent Researcher On Behalf of the University of Parma, Parma, Italy; 3 Unit of Neuroscience, Department of Medicine and Surgery, University of Parma, Parma, Italy; 4 Institut Des Sciences Cognitives Marc Jeannerod, CNRS, Université de Lyon, Bron, France; 5 Maxillo-Facial Surgery Division, Department of Medicine and Surgery, University of Parma, Parma, Italy; 6 Eye Clinic, University of Cagliari, Cagliari, Italy; 7 Institute of Molecular and Clinical Ophthalmology Basel, Basel, Switzerland

**Keywords:** Moebius syndrome, Moebius trait, Epidemiology, Congenital cranial dysinnervation disorders, Strabismus, Congenital facial palsy

## Abstract

**Background:**

The epidemiology of Moebius syndrome (MBS) is difficult to assess. In the present study, we investigated the epidemiology of MBS in a well-defined population within a precise geographical area.

**Materials and methods:**

Our university hospital is the only national referral center for the diagnosis and treatment of MBS. Participants in this cross-sectional study were patients affected by MBS who had been periodically followed by our medical staff since 1998. Most of the patients were referred to our hospital by the Italian Association of Moebius Syndrome (AISMO). Demographic data necessary for study purposes were made available in the AISMO database, updated to April 2018. Subjects were assigned to geographical macroareas that are conventionally used in surveys and epidemiological investigations by the Italian National Institute of Statistics. The rates and prevalence of MBS cases were calculated on the basis of the last available survey of the Italian population. Each study parameter was then calculated with reference to the whole country and macroarea partition. The sex rate and the corresponding prevalence were calculated with respect to the weighted whole population and to the respective sex population. Chi-square analysis was adopted to investigate possible differences among geographical regions and/or sexes. A *p* value < 0.05 was considered statistically significant.

**Results:**

One hundred and sixty-four out of 212 MBS patients fulfilled our inclusion criteria. All cases occurred in Caucasian patients and were sporadic. The median age at diagnosis was 3.6 years, ranging from 0 to 55 years; this range was significantly reduced to 0–5 years (median age at diagnosis: 2.2 years) in patients included after 2007. The calculated prevalence at birth was 0.06 cases per 10,000 live births, with an overall prevalence of 0.27/100,000, without any sex or geographical predominance.

**Conclusions:**

The prevalence of MBS observed herein, rounded for possible underestimation, was 0.3/100,000 people, without any regional difference in the distribution of cases. Our data confirm the rarity of the disease on a national level.

## Introduction

Moebius syndrome (MBS) is considered a congenital cranial dysinnervation disorder (CCDD) [1]. Its clinical features are impaired ocular motility, lagophthalmos, and lack of facial expression; these features are related to congenital, nonprogressive nerve palsy of the 6th and 7th cranial nerves that typically affect newborns bilaterally. MBS is diagnosed according to the "Bethesda Diagnostic Criteria", which have been recently updated to include genetic testing to ascertain a diagnosis [2–5]. The minimum clinical diagnostic criteria for MBS are as follows: “A congenital, uni- or bilateral, nonprogressive facial weakness with limited abduction of the eye(s) and full vertical motility” [2–4]. Patients who do not meet these criteria are labeled “Moebius-like” and are considered affected by a separate congenital disorder. This is of particular importance since the clinical features of MBS overlap with those of many of other CCDDs with well-described genetic bases, such as congenital fibrosis of the extraocular muscles (CFEOM), Duane’s syndrome, and horizontal gaze palsy with progressive scoliosis (HGPPS) [1]. The differential diagnosis of MBS in the early perinatal period may be complex and should consider different neurological disorders that result in an MBS-like phenotype with myopathic facies, abnormalities of the palate and feeding difficulties. To this end, cerebral MRI is a tool to be considered.

At more than a century after the first description of the disease, the etiology of MBS is still unclear; recent studies have postulated a multifactorial pathogenesis in which fetal toxic exposure acts on a genetic predisposition for vascular terminal instability and focal microcirculatory failure in the lower brainstem [6–9]. However, it is not clear what causes these changes and why they specifically disrupt the development of the 6th and 7th cranial nerve nuclei; even less is known about the causes of the extraophthalmological signs and symptoms associated with MBS (e.g., lingual and palate dysfunction, hypoplasia of the hand, clubfoot, and thoracic abnormalities). The exact incidence and prevalence of MBS are not clear; the syndrome is considered a "rare disease", as it affects a very small number of people. Clinicians and researchers estimate that this condition affects 1 in 50,000 to 1 in 500,000 newborns, but this estimate is based on only their personal experience with MBS patients, with no epidemiological basis [3, 9–11]. In a Dutch series, the estimated prevalence of MBS was 0.002% of births (4 per 189,000 newborns); this evidence was obtained in 1996 without the present diagnostic criteria for MBS [12]. The Orphanet Report Series, Rare Disease Collection 2019 reports the estimated prevalence/incidence per 100,000 as "unknown", with only 300 cases described in the literature [13]. Other epidemiological estimates from series reported worldwide are anecdotal, with no statistical basis. It is difficult to plan an epidemiological study of MBS for many reasons: (1) despite the new diagnostic criteria, the disease is often over- and misdiagnosed in newborns; (2) like many other rare diseases, MBS does not have a regional register from which to derive data for epidemiological purposes; (3) there are no referral centers that provide multidisciplinary care with consequent dispersion of cases; (4) few physicians have expertise in MBS, and they may be difficult to reach; and (5) MBS, like other genetic disorders, may carry social stigma, leading affected individuals to self-marginalize. This study reports the epidemiology of MBS in a well-defined population over a very long period; furthermore, we investigated whether there were geographical differences in MBS incidence/prevalence to identify factors that may cause or contribute to its development.

## Methods

Since 1998, the University Hospital of Parma has been designated by the Italian Association of Moebius Syndrome (AISMO, www.moebius-italia.it) and by the Regional Health Department as the only national referral center for the diagnosis and treatment of patients with MBS (e.g., a multidisciplinary facility treating conditions ranging from strabismus correction to smile surgery). This allowed us to contact and virtually follow all of the MBS patients living in Italy. Even MBS patients who currently received medical care elsewhere were evaluated in our hospital at least once during the diagnostic confirmatory phase. All medical data are regularly updated and preserved in the AISMO database, which serves as the electronic medical records of these patients, based on a specific agreement between our university hospital and the association since 1999. Any additional relevant information, such as pregnancy with possible consumption of drugs, type of delivery, and details of the medical history of MBS relatives, is also recorded in the AISMO register. The database is available to only approved researchers and is accessible for medical or scientific purposes regarding MBS only.

As previously reported, the MBS cases included in our analysis satisfied the updated “Bethesda Diagnostic Criteria”. The exclusion criteria included the finding of a genetic profile associated with MBS-like myopathic facies (in particular alterations in the *TUBB3, HOXA1, HOXB1, ROBO3* genes) [14] and the possible finding of karyotype macroanomalies producing myopathic facies (e.g., a trisomy of the chromosome 18 or 3). Data on genetic testing or Moebius-like symptoms in relatives and parental consanguinity were obtained from the AISMO database, which acts as a multidisciplinary medical chart and is regularly updated with key information. One patient in our present analysis had already been included in a prior series (patient #11) and carries a pathogenic variant in the *REV3L* gene [15].

Every MBS patient was periodically evaluated by our multidisciplinary team of physicians, which includes an ophthalmologist (A.C.), a neonatologist or pediatrician, a speech therapist, an orthodontist, an orthopedist (for children with clubfoot or finger anomalies), and a maxillofacial surgeon with expertise in smile surgery; the frequency of visits depended on the severity of the disease, usually ranging from 6 to 24 months. Each visit included a comprehensive ophthalmological evaluation of extraocular motility and refraction under cycloplegia for pediatric patients. A detailed history was obtained from relatives of each patient at the first visit and updated at every visit. All patients (or their relatives if minors) had previously given AISMO consent to use their demographic data for research purposes and statistical analysis. The present series also includes patients who had been included in previous research papers [2, 16]. The study adhered to the tenets of the Declaration of Helsinki and was approved by the local ethics committee (No. 93/2019/OSS*/UNIPR/June 14, 2019).

### Statistical analysis

The authors used data made available by AISMO to obtain the following information for each registered member: date of birth, date and age at diagnosis, sex, and place of provenance/residence. The subjects were assigned to five geographical regions (i.e., northeast, northwest, central, south, and islands) conventionally used in surveys and epidemiological investigations in Italy. Every study parameter was then calculated with reference to the entire country and each region. The rates and prevalence (number of cases per 100,000 people) of MBS were calculated referring to the 2018 Italian census performed by the National Institute of Statistics [16]. The rate and corresponding prevalence were calculated for the entire population and each sex (i.e., affected males/Italian males; affected females/Italian females). The prevalence at birth was calculated by referring to the year of birth for each patient. Chi-square (χ^2^) analysis was used to investigate possible differences among geographic regions and between sexes. For sex comparisons, unweighted (i.e., each rate was weighted equally across regions, independent of the actual sex population in the corresponding region) and weighted (i.e., each rate was scaled to the corresponding sex weight according to the population density in each region) data were analyzed. A *p* value < 0.05 was considered statistically significant.

### Data availability statement

Data used for the study can be accessed at the following links: www.moebius-italia.it; and www.istat.it.

## Results

### Descriptive statistics

The AISMO database contained 231 subjects. Of these, 59 subjects did not fully satisfy the “Bethesda Criteria” or they had genetic profiles not compatible with MBS; 4 subjects were not Italian citizens, and 4 subjects had incomplete data. In total, 67 subjects (29%) were excluded from our cohort. The remaining 164 MBS patients (73 men, 44.5%) were included in the study and analyzed for epidemiological purposes (Table 1).

**Table 1:** Relative rate (%) of MBS in the five Italian regions

	No. of cases (N)	Age range at diagnosis (years)	No. of males (M)	% Males	No. of females (F)	% Females
Northeast	35	0*–38	16	45.7	19	54.3
Northwest	51	0*–55	19	37.3	32	62.7
Central	34	0*–49	16	47.1	18	52.9
South	29	0*–34	15	51.7	14	48.3
Islands	15	0*–18	7	46.7	8	53.3
Total	164	0*–55	73	44.5	91	55.5

0* means that the diagnosis was made within the first year of life in newborns

All patients were Caucasian, and all cases were sporadic. The median age at diagnosis was 3.6 years, ranging from 0 to 55 years (a value of 0 means that the diagnosis was made within the first 12 months of life); this range was significantly reduced to 0–5 years (median age at diagnosis: 2.2 years) for patients included after 2007.

**Fig. 1:**
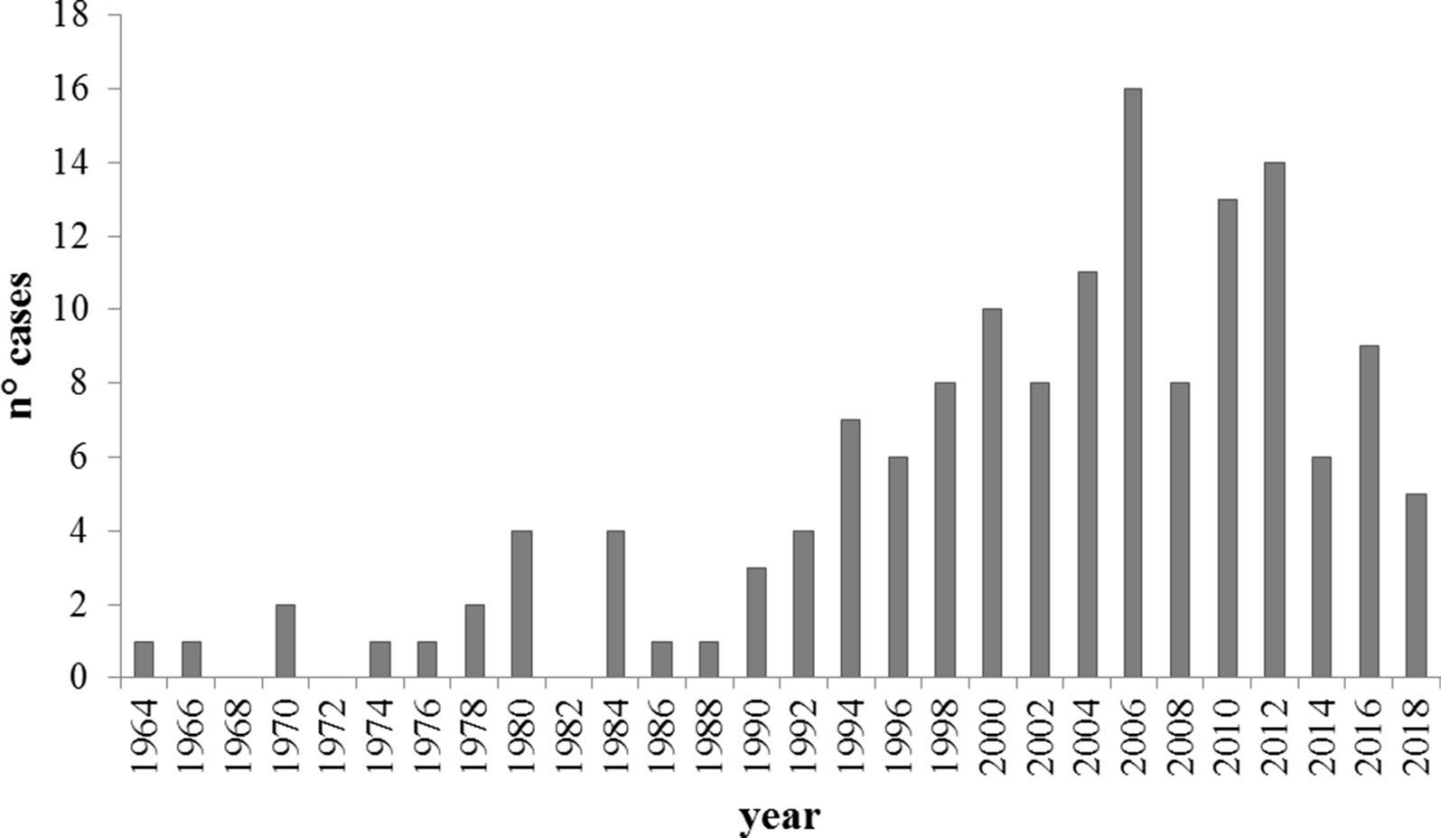
Number of newly recorded MBS cases in each biennium from 1964 to 2018. Cases registered in odd years were assigned to the next even year

Figure 1 shows the newly recorded cases in the AISMO register based on the year of birth; a progressive increase in recorded cases is evident after 1998, when the AISMO register was established, with new diagnoses peaking (16 cases) in 2005–2006 (coincidentally after the Consensus Conference on Moebius Syndrome, which produced the “Bethesda Criteria”). The birth prevalence calculated using the most recent available national data was 0.06 cases per 10,000 live births (in 2016, three newborns out of 473,438 live births were diagnosed with MBS in Italy according to the database of the Istituto Nazionale di Statistica: https://www.istat.it). The mortality rate in our cohort was 0 (zero), as there were no known deaths. In our series, we recorded a family with two monozygotic twins who were both affected but had different clinical manifestations of the disease; we had no cases of affected siblings, and almost all cases were bilateral (8 cases were bilateral but asymmetric and 4 were monolateral). The extraocular-associated features in our patients are summarized in Table 2.

**Table 2:** Percentages (%) of extraocular anomalies in our MBS patients (it was possible to have more than one in a single patient)

Extraocular anomalies	%
Club foot	32
Tongue dysfunctions, including suction defects and dysphagia	24
Speech problems	22
Hypoplastic hand	20
Dental anomalies	18
Palate malformations	7
Hearing deficiency	7
Scoliosis	5
Developmental, cognitive, or behavioral delay	4
Poland's syndrome	2

### Statistical analysis

**Fig. 2:**
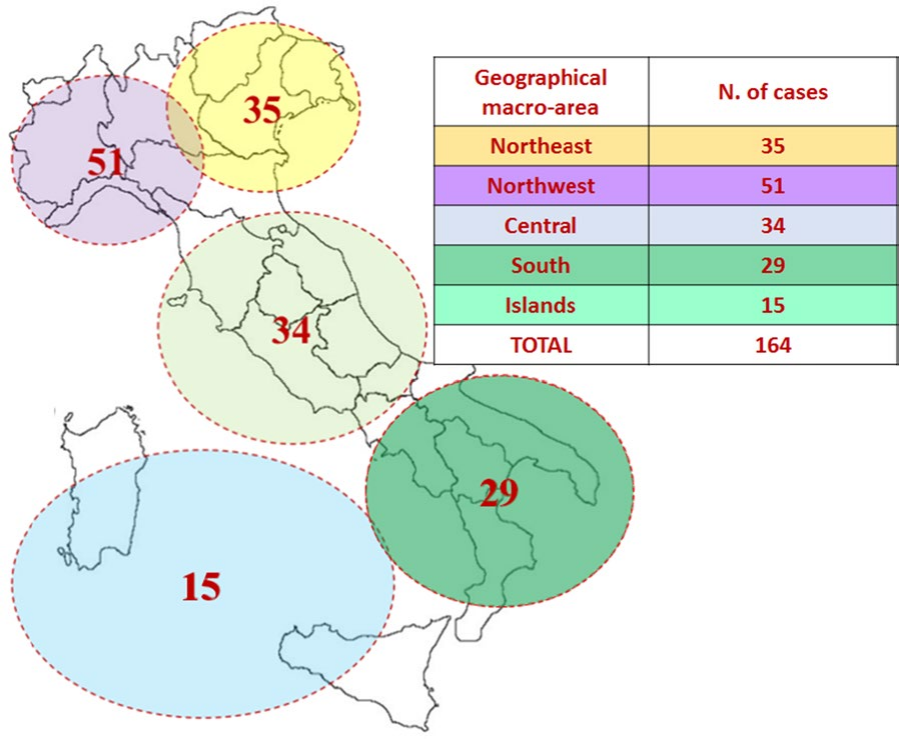
The prevalence of MBS (no. of cases/100,000 people) was determined for each geographic area (Table 2), with an overall prevalence of 0.27/100,000

The relative rate of MBS in the Italian population was calculated for males and females in terms of the total number of patients diagnosed and the number diagnosed in each region (Table 1). The rates were evenly distributed across the different regions, with the exception of the northwest, where more cases were located (Fig. 2). Moreover, in this region, the sex ratio differed since there were 1.5 times more females than males (62.7% vs. 37.3%). The difference in the rate in the northwest was confirmed with reference to both the total population (P-MT: 0.12 vs. P-FT: 0.20) and sex-based subanalyses (P- MM: 0.24 vs. P-FF: 0.39) (see Table 3).

**Table 3:** Prevalence (including sex-specific) of MBS in each region

	P-TT Tot cases/Pop^a^ (per 100,000)	P-MT M cases/Pop^a^ (per 100,000)	P-FT F cases/Pop^a^ (per 100,000)	P-MM M cases/M-Pop^b^ (per 100,000)	P-FF F cases/F-Pop^c^ (per 100,000)
Northeast	0.30	0.14	0.16	0.28	0.32
Northwest	0.32	0.12	0.20	0.24	0.39
Central	0.28	0.13	0.15	0.27	0.29
South	0.21	0.11	0.10	0.22	0.19
Islands	0.22	0.10	0.12	0.22	0.23
Total	0.27	0.12	0.15	0.25	0.29

^a^Pop: overall Italian population size

^b^M-Pop: Italian male population size

^c^F-Pop: Italian female population size

The MBS rates of the total population (i.e., both males and females) stratified into five regions differed significantly (*p* < 0.001; χ^2^-test). To verify whether this result was due to the larger population in the northwest, the data were weighted to account for the different populations in the five regions. This analysis involved weighted rate data (i.e., data rescaled to the actual population in each region). After this adjustment, no statistical significance was observed. Finally, a χ^2^-test to compare the rates between sexes was performed. The analysis of males confirmed that the distributions across the different regions were similar (no significant differences for both "unweighted" and "weighted" data in the male populations in the different regions). The analysis of females showed a significant (*p* < 0.001) difference for "unweighted" data. However, this difference lost significance when "weighting" was applied to the female populations in different regions. An analysis of the total weighted population dataset showed no significant differences among the five regions.

## Discussion

This study examined the epidemiology of Moebius syndrome in a well-defined population within a precise geographical area using definite diagnostic criteria for MBS. We found that the overall prevalence of MBS was 0.27/100,000 newborns. This value can be reasonably rounded to 0.3/100,000 (0.0003%) because few patients with MBS may have missed appointments with our medical staff and/or were not enrolled in the AISMO register. This may occur when dealing with patients/families affected by congenital genetic disorders, which may carry social stigma leading to self-marginalization. Regardless, the prevalence observed in our study was different from data reported in different parts of the world. For example, in a Dutch series, Verzijl et al. in 1996 estimated a prevalence of 0.002% (i.e., approximately ten times higher than our results) for MBS, but the details about their sources were poor and different diagnostic criteria were used with respect to those presently adopted [13]. Similar relatively high prevalence rates were reported by physicians with expertise in MBS in the United States, Sweden, and Brazil but without any population-based analysis [9–11]. Based on our data, we can confirm that MBS is an extremely rare disease. Furthermore, our data may be considered if a dedicated national healthcare program is planned or reorganized. Another important consideration is that we found a uniform distribution of MBS cases among the five areas considered. These five regions were conceived by ISTAT, as people living in them have different social, economic, and working lifestyles, with different climates characterizing each region. As we did not identify a region with a higher prevalence of MBS cases, we can exclude environmental factors such as pollution; weather conditions, such as intense cold or heat; and prolonged sun exposure during pregnancy as causes of MBS. It appears that the environment had little or no influence on disease pathogenesis in our population. The only reported factor that significantly increased the risk of newborns being affected by MBS (by a factor of 30) is the use of misoprostol during the first trimester of pregnancy [19]. Misoprostol (a synthetic prostaglandin E analog) is an illegal abortifacient widely used in Brazil and other countries in South and Central America.

As misoprostol is not administered in Italy, our epidemiological data on MBS lack any pharmacological bias, at least as far as misoprostol is concerned. In addition to misoprostol use, based on the electronic medical chart of each patient made available by the AISMO register, we also excluded the use of vasoconstrictor agents and cocaine and the presence of abdominal trauma during pregnancy for all the considered patients. In our series, the disease affected males and females equally, thus supporting the evidence that MBS is not an inherited X- or Y-related disease. Moreover, all included cases were “sporadic” (i.e., no affected siblings and no family history of MBS). This evidence is in line with the recent hypothesis that MBS has a multifactorial basis with genetic mechanisms having a predisposing role [7–9, 15, 18]. However, we have strongly supported genetic counseling for every family with an affected member. This with the purpose of investigating the possible transmission of unknown Moebius-like traits. In these families, the risk of recurrence in offspring has been found to reach 50% [20]. Furthermore, genetic testing may be important for a deeper understanding of the pathogenesis of forms presenting with extraocular-associated anomalies.

Another interesting point is that patients who were evaluated by our staff after 2007 received an earlier diagnosis than those born before 2007 (2.2 *vs*. 3.4 years, respectively), with a significant reduction in the range, which was decreased to between 0 and 5 years of age. This finding likely resulted from efforts made during the last two decades by international associations to increase knowledge of this disease; another explanation may be that our specialized medical staff can be contacted easily by relatives of affected newborns, thereby allowing an earlier diagnosis. An early diagnosis of MBS means that we can initiate care for affected individuals at a young age. This can have extremely positive effects on patients' quality of life by significantly reducing the behavioral and psychological problems related to MBS [21]. For example, with an early diagnosis, we can plan smile surgery at preschool age or we can perform early strabismus surgery when indicated, thereby improving visual performance and reducing the risk of amblyopia.

## Conclusion

Our data add to the knowledge of MBS, providing epidemiological information from a highly populated European country, which may be particularly useful when devising medical policies regarding this rare disease. Most rare diseases are considered "orphan diseases" and have no effective treatments; people affected are often psychologically, socially, economically, and culturally vulnerable, as they receive no treatment for their medical condition. These difficulties can be overcome, and the efforts made by the scientific community can increase our knowledge and provide new hope for the future treatment of this disorder.

## Data Availability

Data used for the study can be checked at the following links: www.moebius-italia.it; and www.istat.it.

## Abbreviations

MBS: Moebius Syndrome
AISMO: Italian Association of Moebius Syndrome
